# Clinical analysis and functional characterization of KCNQ2-related developmental and epileptic encephalopathy

**DOI:** 10.3389/fnmol.2023.1205265

**Published:** 2023-07-11

**Authors:** Jia Ye, Siyang Tang, Pu Miao, Zhefeng Gong, Qiang Shu, Jianhua Feng, Yuezhou Li

**Affiliations:** ^1^National Clinical Research Center for Child Health, The Children's Hospital, Zhejiang University School of Medicine, Hangzhou, China; ^2^Pediatric Department, Second Affiliated Hospital, Zhejiang University School of Medicine, Hangzhou, China; ^3^School of Brain Science and Brain Medicine, Zhejiang University School of Medicine, Hangzhou, China

**Keywords:** developmental and epileptic encephalopathy, dominant-negative effects, developmental delay, KCNQ2, pediatrics

## Abstract

**Background:**

Developmental and epileptic encephalopathy (DEE) is a condition characterized by severe seizures and a range of developmental impairments. Pathogenic variants in *KCNQ2*, encoding for potassium channel subunit, cause *KCNQ2*-related DEE. This study aimed to examine the relationships between genotype and phenotype in *KCNQ2*-related DEE.

**Methods:**

In total, 12 patients were enrolled in this study for genetic testing, clinical analysis, and developmental evaluation. Pathogenic variants of KCNQ2 were characterized through a whole-cell electrophysiological recording expressed in Chinese hamster ovary (CHO) cells. The expression levels of the KCNQ2 subunit and its localization at the plasma membrane were determined using Western blot analysis.

**Results:**

Seizures were detected in all patients. All DEE patients showed evidence of developmental delay. In total, 11 *de novo* KCNQ2 variants were identified, including 10 missense variants from DEE patients and one truncating variant from a patient with self-limited neonatal epilepsy (SeLNE). All variants were found to be loss of function through analysis of M-currents using patch-clamp recordings. The functional impact of variants on M-current in heteromericKCNQ2/3 channels may be associated with the severity of developmental disorders in DEE. The variants with dominant-negative effects in heteromeric channels may be responsible for the profound developmental phenotype.

**Conclusion:**

The mechanism underlying *KCNQ2*-related DEE involves a reduction of the M-current through dominant-negative effects, and the severity of developmental disorders in DEE may be predicted by the impact of variants on the M-current of heteromericKCNQ2/3 channels.

## Introduction

Voltage-gated potassium channels Kv7.2 encoded by the *KCNQ2* gene have been recognized as a common genetic cause of epileptic disorders (Lerche et al., 2001; Armijo et al., 2005; Errington et al., 2005). The subunit Kv7.2 combines with the KCNQ3 subunit to form a heterotetrameric potassium channel that underlies the M-current (Brown and Adams, 1980; Wang et al., 1998; Shapiro et al., 2000). The M-current plays a critical role in controlling the threshold of neuronal excitability by stabilizing the resting potential and suppressing the repetitive firing of action potentials (Delmas and Brown, 2005; Tzingounis and Nicoll, 2008). Pathogenic variants in KCNQ2 have been linked to a range of neonatal epileptic disorders from self-limited neonatal epilepsy (SeLNE) to more severe neonatal-onset developmental and epileptic encephalopathy (DEE) (Kato et al., 2013). SeLNE is characterized by seizures that occur in the first days of life but typically disappear spontaneously after a few weeks to months (Steinlein et al., 2007; Maljevic and Lerche, 2014; Millichap et al., 2016). DEE, on the other hand, is characterized by severe seizures, developmental delay, and a poor prognosis (Berg et al., 2021; Specchio and Curatolo, 2021). Furthermore, a recent study has indicated that KCNQ2 may be linked to intellectual disability in the absence of epilepsy (Mary et al., 2021).

The relationship between genotype and phenotype in KCNQ2-related epilepsy is not fully understood. However, patch-clamp recordings have improved our understanding of KCNQ2 channel dysfunction and the spectrum of clinical severity (Miceli et al., 2013; Soldovieri et al., 2014; Vanoye et al., 2022). SeLNE is due to haploinsufficiency as the majority of SeLNE variants have only a subtle loss-of-function (LOF) effect on the current amplitude of expressed heteromeric channels, leading to a small reduction in current (Schroeder et al., 1998). Most variants associated with DEE lead to a more significant reduction in total current, suggesting a dominant-negative effect as the primary pathogenic mechanism for the severe epilepsy phenotype (Orhan et al., 2014; Gomis-Perez et al., 2019). The recent studies on a mouse model carrying the DEE variant p.T274M showed that spontaneous seizures occurred more frequently at postweaning stages than at juvenile stages and were associated with reduced M-current density and hyperexcitability of pyramidal cells in motor cortical slices (Milh et al., 2020; Biba-Maazou et al., 2022). However, cognitive development and behavior are often impaired in most patients with DEE, even if epilepsy is well-controlled. In these cases, controlling seizures does not necessarily lead to good cognitive outcomes (Berg et al., 2021; Specchio and Curatolo, 2021). The question of whether KCNQ2 variants are functionally associated with the developmental impairment of DEE and how to predict prognosis remains unanswered.

In this study, we identified 11 *de novo* KCNQ2 variants including 10 missense variants from DEE patients and one truncating variant from a SeLNE patient. Combining clinical analysis and whole-cell electrophysiological recording of M-currents resulting from homomericKCNQ2 or heteromericKCNQ2/3 channels, we investigated the functional consequence of pathogenic variants that were correlated with developmental impairment. Our findings expanded the variants associated with the spectrum of clinical/functional diversity and provided insights into the phenotype–genotype relationships of KCNQ2-related DEE.

## Materials and methods

### Patients

A total of 12 patients including eight female patients and four male patients diagnosed in the Second Affiliated Hospital, Zhejiang University School of Medicine, with neonatal-onset epilepsy were enrolled in this study. No underlying non-genetic factors, such as acquired brain injury, were identified in any of the participants. Out of the 12 patients, 11 exhibited mild-to-profound developmental delay, with only one patient exhibiting normal development. Clinical data including demographic information, seizure history, developmental history, EEG results, cranial imaging results, and treatment information were collected and evaluated by two pediatric neurologists. Developmental milestones, such as eye contact, head control, sitting, standing, walking, and language ability, as well as neurologic and behavioral features, were assessed using the composite neurodevelopmental score system of *STXBP1*-DEE for patients who were at least 3 years of age at the time of the study (Balagura et al., 2022). Genetic sequencing was performed, and the pathogenicity of the identified variants was evaluated by a clinical geneticist according to the guidelines of the American College of Medical Genetics.

### Mutagenesis and heterologous expression of KCNQ2

The wild-type (WT) KCNQ2 was subcloned into a pIRES2-EGFP vector, and the variants were introduced using a ClonExpress II One Step Cloning Kit (vazyme). KCNQ3 was subcloned into a pIRES2-mCherry vector. The ORFs of all plasmids were confirmed by sequencing full length before transfection. Channel subunits were expressed in Chinese hamster ovary (CHO) cells by transient transfection. CHO cells were maintained in DMEM (Gibco), containing 10% fetal bovine serum (Gibco), penicillin (100 U/ml), and streptomycin (100 μg/ml) at 37°C with 5% CO_2_. For electrophysiological experiments, the cells were seeded on glass coverslips and transfected on the next day with a certain ratio of plasmids using Lipofectamine 2000; the total cDNA in the transfection mixture was kept constant at 3 μg. Transfection-positive cells were identified by the fluorescent protein and were used for whole-cell patch-clamp recording.

### Cell surface biotinylation and Western blotting

The expression of KCNQ2 subunits in CHO cells was investigated as previously described (Maljevic et al., 2011; Miceli et al., 2015). After 24 h of transfection, the CHO cells were treated with Sulfo–NHS–LC–Biotin (Thermo) following the manufacturer protocol and then lysed. The cell lysates were then reacted with Streptavidin UltraLink Resin (Thermo). The channel subunits in total lysates and streptavidin precipitates were analyzed by Western blotting using rabbit monoclonal anti-KCNQ2 primary antibodies (D9L5S, dilution 1:1,000; Cell Signaling Technology, 14752), followed by secondary antibodies (Alexa Fluor 680 Conjugate; dilution 1:5000; Abcam, 175773). An anti-beta actin antibody (dilution 1:5,000; GenScript) was used to check for equal protein loading.

### Electrophysiology

Macroscopic currents were recorded at room temperature (20–22°C) 1 day after transfection with an Axon MultiClamp 700B amplifier (Axon Instruments). Patch pipettes were pulled to a pipette resistance of 3–5MΩ. The pipette solution (intracellular) contained 140 mM KCl, 2 mM MgCl_2_, 10 mM EGTA, 10 mM HEPES, and 5 mM Mg-ATP, and the pH was adjusted to 7.4 with KOH. The bath solution contained 138 Mm NaCl, 2 mM CaCl_2_, 5.4 mMKCl, 1 mM MgCl_2_, 10 mM d-(+) glucose, and 10 mM HEPES, and the pH was adjusted to 7.4 with NaOH. Current densities were calculated as peak K+ currents at +40 mV. To generate conductance–voltage (G/V) curves, the cells were held at −80 mV and then depolarized for 1.5 s from −80 to +50 mV using an incremental pulse of 10 mV, followed by an isopotential pulse at −10 mV of 400 ms. The current values recorded at the beginning of the−10 mV pulse were normalized and fitted using the Boltzmann function: y = max/[1 + exp(V _0.5_ - V)/k], to obtain the half-maximum activation voltage (V_0.5_) and the slope factor (k).

### Statistics

All data were expressed as mean ± SEM, and statistically significant differences between the data were evaluated with one-way ANOVA by Bonferroni *post hoc* test, with the threshold set at a *p*-value of <0.05.

## Results

### Clinical features

A total of 12 patients, consisting of eight female patients and four male patients, were included in this study (see Table 1 for detailed clinical features). All patients were born without any acquired brain injury, such as those related to encephalitis, hypoxia, neoplasm, metabolic disturbance, traumatic brain injury, or toxicity.

**Table 1:** Clinical features of patients.

	**Patient 1**	**Patient 2**	**Patient 3**	**Patient 4**	**Patient 5**	**Patient 6**	**Patient 7**	**Patient 8**	**Patient 9**	**Patient 10**	**Patient 11**	**Patient 12**
Sex	Female	Female	Female	Male	Female	Female	Male	Female	Female	Male	Female	Male
Age	3y	3y 9m	5m	4y 2m	3y	5y	5y	died at 2y	3y	died at 2m	3y	2y 2m
Variants	c.553G>A p.A185T^a^	c.643G>A p.G215R^b^	c.712A>C p.I238L^a^	c.794C>T A265V^c^	c.829A>G p.T277A^d^	c.853C>T p.P285S^d^	c.868G>A; p.G290S^e^	c.901G>A; p.G301S^f^	c.901G>A; p.G301S^f^	c.1687G>A; p.D563N^g^	c.1734G>A; p.M578I^h^	c.1816A>T; p.K606X^a^
Location	S3	S4-S5	S5	Pore loop	Pore loop	Pore loop	S6	S6	S6	C-term	C-term	C- truncating
Inheritance	De novo	De novo	De novo	De novo	De novo	De novo	De novo	De novo	De novo	De novo	De novo	De novo
Seizure onset	2d	14d	1d	2d	1d	1d	3d	1d	7d	1d	7d	3d
Seizure types	Tonic	Spasms, focal, GTCS	Focal, GTCS	Tonic, spams	Tonic, spasms, focal	Tonic, spasms, focal	Tonic	Tonic	Tonic	Tonic	Tonic, spams	Tonic
EEG	BUS	BUS->H	BUS	BUS->H	BUS->H	BUS->H	BUS	MS	BUS	MS	BUS->H	Normal
Diagnosis	OS	OS->IS	OS	OS->IS	OS->IS	OS->IS	OS	EIMFS	OS	EIMFS	OS->IS	SeLNE
ASMs used	PB, LEV	PB, TPM, LEV, VGB, OXC, ACTH, LEV, CZP, CLB, LCM	LEV, OXC	PB, VPA	LEV, PB, TPM, NZP, OXC, CLB, LCM, CBZ	PB, TPM, VPA, NZP, M	PB, TPM, OXC,	OXC, VPA	VPA, TPM, CZP, CBZ	PB, TPM, OXC, NZP	TPM, VPA, OXC	PB, LEV, TPM
Effective ASMs	LEV	LCM	OXC	VPA	OXC, LCM	/	OXC	OXC	CBZ, TPM	OXC	OXC	TPM
Seizure free	6 m	9 m	3 m	3 m	6 m	9 m	1.5 m	Died	5 m	Died	8 m	2 m
Developmental delay	Head control, eye contact walking, and no language	Poor eye contact, no head control, and hypotonia	Poor head Control and poor eye contact	No eye contact, no head control, difficulty in swallowing, and hypotonia	Poor head control, poor eye contact, unable to sit, and hypotonia	Head control, unable to sit, poor eye contact, and hypotonia	Poor head control, poor eye contact, unable to sit, and pyramidal signs	Died at 2 y due todysphagia and asphyxia	Poor eye contact, no head control, and hypotonia	Died at 2 m due to dysphagia and asphyxia	Head control, eye contact, walking, no language, and hypotonia	normal
Composite developmental Score^*^	6	2	/	1.5	2.5	3	2.5	/	1.5	/	5.5	10
Developmental ratings	Moderate	Profound	/	Profound	Profound	Severe	Profound	/	Profound	/	Moderate	Normal

a Mutations are new;

b Dalen Meurs-Van der Schoor et al., 2014;

c Weckhuysen et al., 2012;

d Miao et al., 2018;

e Milh et al., 2013;

f Parrini et al., 2017;

g Milh et al., 2015;

h Numis et al., 2014. ACTH, adrenocorticotropic hormone; ASMs, antiseizure medications; BUS, burst-suppression; CBZ, carbamazepine; CLB, clobazam; CZP, clonazepam; EIMFS, epilepsy of infancy with migrating focal seizures; GTCS, generalized tonic–clonic seizure; H, hypsarrhythmia; IS, infantile spasms; LCM, lacosamide; LEV, levetiracetam; M, methylprednisolone; MS, Multiple spikes; NZP, nitrazepam; OS, Ohtahara syndrome; OXC, oxcarbazepine; PB, phenobarbital; SeLNE, Self-limited neonatal epilepsy; TPM, topiramate; VGB, vigabatrin; VPA, valproic acid. ^*^According to STXBP1 composite developmental score (>3 y): mild ≥7, moderate: ≥5 and < 7, severe: ≥3 and < 5, and profound: < 3.

Patients 1–11 were detected with DEE phenotypes, while patient 12 was diagnosed with SeLNE. All the patients had neonatal-onset epilepsy, with onset ranging from 1 day to 14 days after birth. Seizure onset within the first 3 days of life was observed in nine patients. Tonic seizures were found in nearly all patients as the initial seizure type, with burst suppression and multiple spikes being the most common EEG patterns at the early stage of the disease.

Although seizures were controlled within 3–9 months, all DEE patients exhibited varying degrees of developmental delay, most of them (>3 years old) being followed up with profound developmental delay by using the composite neurodevelopmental score system of DEE patients. Developmental delay became the primary symptom after 1 year of age in all DEE patients, and no drugs or other therapies were found to improve the impairments. The SeLNE patient presented seizure-free 2 months after birth and demonstrated normal subsequent development.

Two DEE patients died during the follow-up period. Patient 10 had tonic seizure onset 8 h after birth, which was frequently refractory to multiple antiseizure medications (ASMs). At 2 months of age, he had difficulty swallowing milk and died from suffocation by choking. Patient 8 had tonic seizure onset 1 day after birth and was also refractory to multiple ASMs. At 2 years old, she suffered frequent vomiting and developed food intolerance gradually, leading to her death from suffocation by choking.

In all patients, seizures were found to be drug resistant to multiple ASMs, either used alone or in combination. Valproate (VPA) and topiramate (TPM) were the most frequently used ASMs in this study, followed by phenobarbital (PB), levetiracetam (LEV), and oxcarbazepine (OXC). Of these, OXC was the most effective drug, effectively controlling seizures in five out of six patients, followed by TPM (two out of eight patients) and LEV (one out of six patients). PB was used in eight patients but was found to be ineffective in each case. After unsuccessful trials of multiple ASMs, lacosamide (LCM) was effectively used in patients 2 and 5. PB, TPM, VPA, nitrazepam (NZP), and methylprednisolone (M) were tried in patient 6, but none were found to be effective. Her seizures resolved spontaneously at the age of 9 months.

### Genetic analysis

Genetic analysis revealed 11 *de novo* KCNQ2 variants including 10 missense variants from DEE patients and one truncating variant from SeLNE patients. The variants identified include (NM_172107.4) the following: c.553G>A (p.A185T) in Patient 1; c.643G>A (p.G215R) in Patient 2; c.712A>C (p.I238L) in Patient 3; c.794C>T (p.A265V) in Patient 4; c.829A>G (p.T277A) in Patient 5; c.853C>T (p.P285S) in Patient 6; c.868G>A (p.G290S) in Patient 7; c.901G>A (p.G301S) in Patients 8 and 9; c.1687G>A (p.D563N) in Patient 10; c.1734G>A (p.M578I) in Patient 11; and c.1816A>T (p.K606X) in Patient 12.

Among these variants, p.G215R, p.A265V, p.T277A, p.P285S, p.G290S, p.G301S, and p.M578I have been previously reported in patients with neonatal seizures and developmental delay but without functional characterization (Weckhuysen et al., 2012; Milh et al., 2013; Dalen Meurs-Van der Schoor et al., 2014; Numis et al., 2014; Parrini et al., 2017; Miao et al., 2018). P.D563N has been previously reported in a case report and functionally characterized in an article (Milh et al., 2015; Ambrosino et al., 2018). The remaining variants (p.A185T, p.I238L, and p.K606X) have not been reported previously.

**Figure 1:**
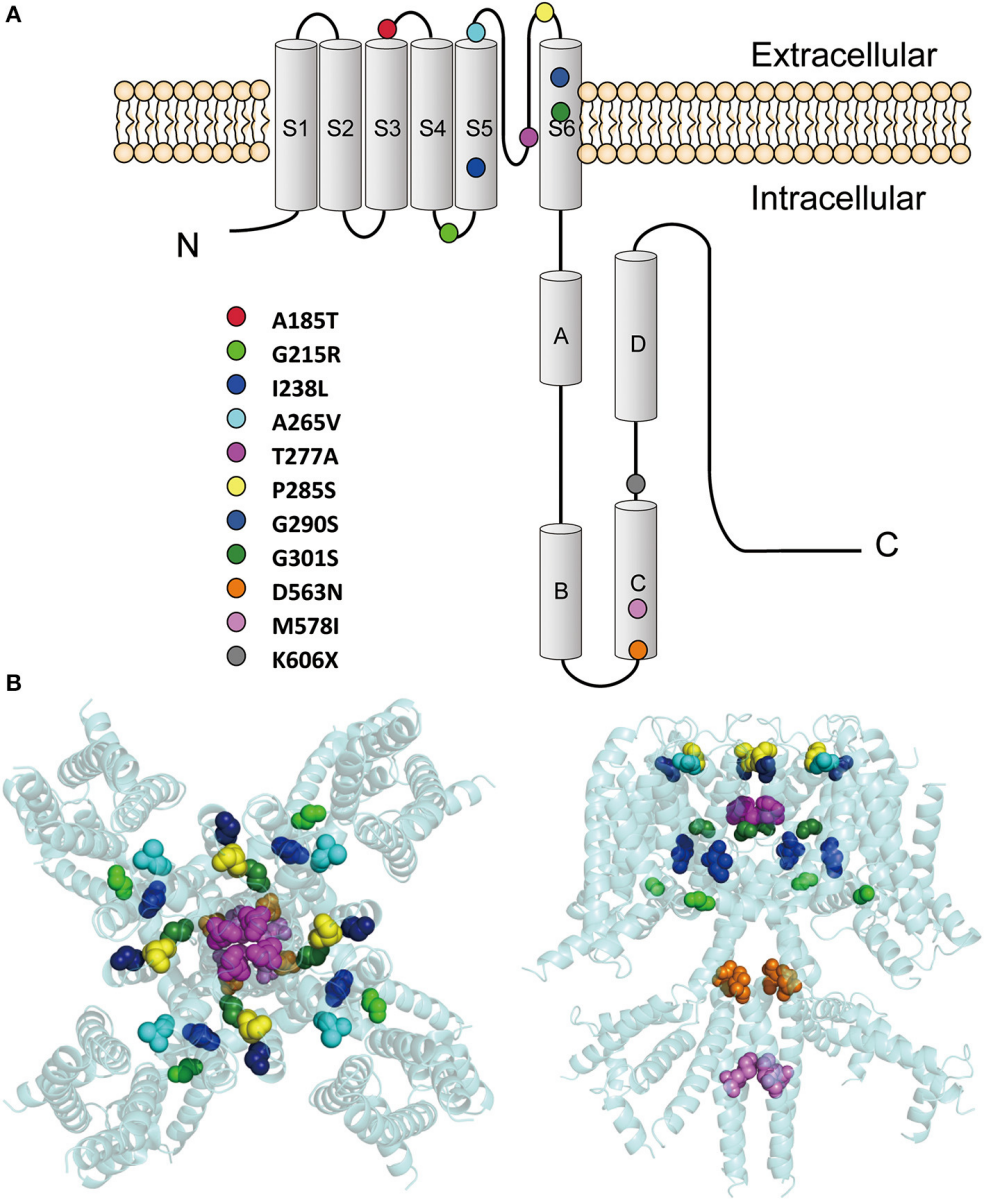
Locations of the pathogenic KCNQ2 variants. **(A)** Topology diagram of the KCNQ2 channel. The KCNQ2 subunits possess six transmembrane segment regions (S1-S6) and a long intracellular C terminus. Segments S1–S3 and S4 form the voltage-sensing domain. S5–S6 in conjunction with their extracellular linker constitute the channel pore. C-terminal helices A and B indicate the sites for interaction with calmodulin. Helices C and D are involved in subunit–subunit interactions. The locations of the variants described in the present report are shown as circles in different colors. **(B)** Structure of the human KCNQ2 (PDB:7CR3) (Li et al., 2021). The side view is shown on the left and the top view is on the right. The variants are shown as sphere styles in different colors. The p.A185T and p.K606X are not shown because they are not resolved in the original structure.

These variants are located throughout the KCNQ2 channel (Figure 1), with one located in the S3-S4 linker (p.A185T), one in the S4-S5 linker (p.G215R), six in the pore-forming S5-S6 region (p.I238L, p.A265V, p.T277A, p.P285S, p.G290S, and p.G301S), and three in the C terminus (p.D563N, p.M578I, and p.K606X). The p.K606X variant creates a stop code and results in a truncated channel with a partial deletion from K606 in the C terminus.

### Functional studies

**Figure 2:**
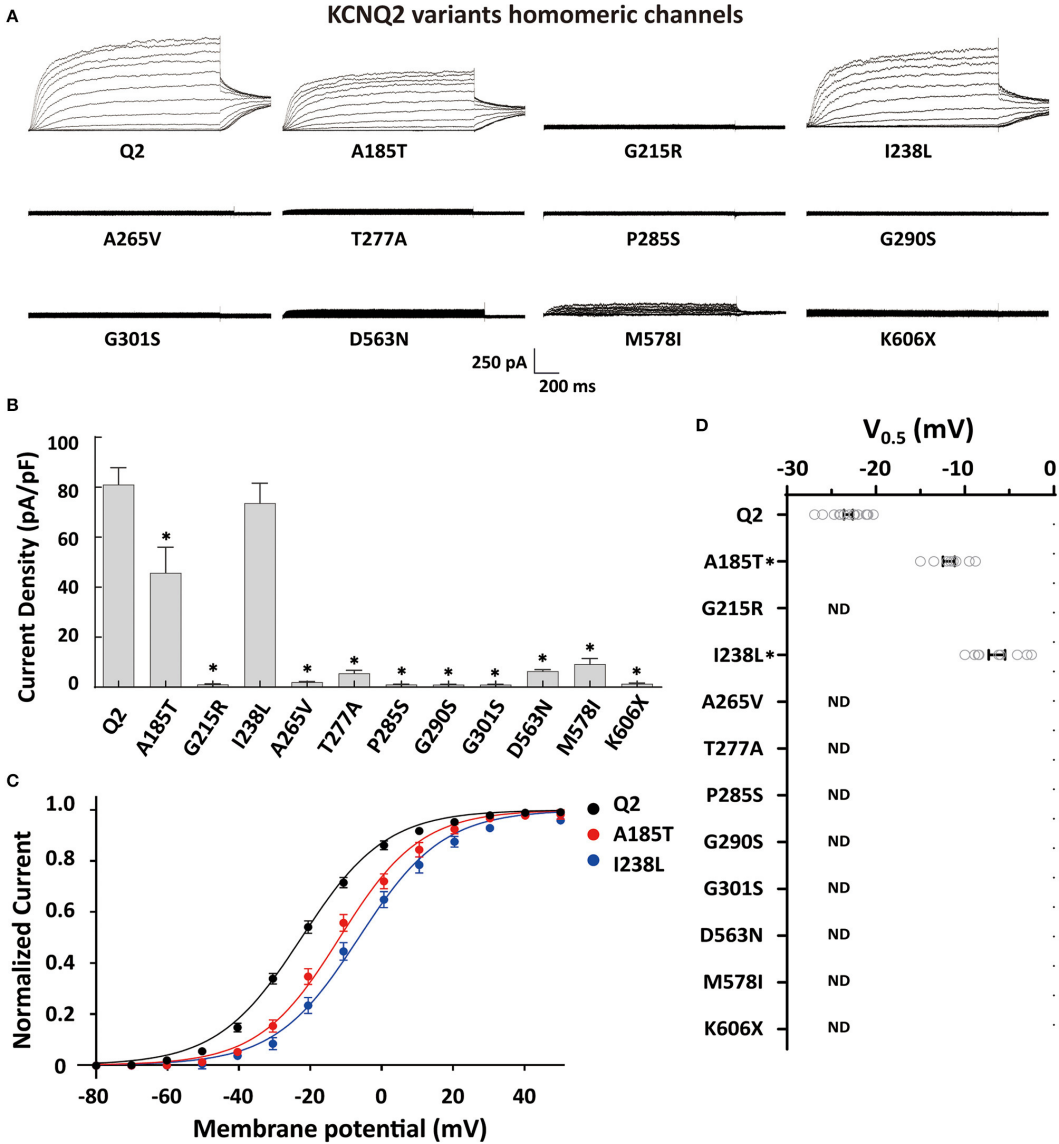
Functional properties of the homomericKCNQ2 variants. **(A)** Macroscopic current traces recorded in CHO cells transfected with KCNQ2 variants (3μg) in homomeric configuration. Current scale, 250 pA; time scale, 200 ms. **(B)** Mean current densities of KCNQ2 channel at +40 mV in the homozygous state. **(C)** Current–voltage relationships of the KCNQ2 homomeric channel were determined from tail current amplitudes. Lines represent fits of a Boltzmann function. **(D)** Differences in activation of V_0.5_ were determined for the KCNQ2 channel expressed in the homozygous state (*n* = 8–15). Statistically significant differences *p < 0.05 versus WT KCNQ2.

To determine the effects of the variants on the KCNQ2 channel, we conducted whole-cell recordings on transiently expressed WT and mutated KCNQ2 channels in CHO cells. The results showed that the homomeric WT KCNQ2 channels generated potassium-selective currents that activated at a threshold potential of around −50 mV and did not inactivate. However, the variants p.G215R, p.A265V, p.T277A, p.P285S, p.G290S, p.G301S, p.D563N, p.M578I, or p.K606X showed barely detectable or negligible currents (Figure 2A). The variant p.A185T resulted in reduced current amplitudes, while p.I238L showed 91% current density compared to WT (Figure 2B). Both p.A185T and p.I238L significantly shifted the activation curve to more positive potentials than WT (Figures 2C, D). The results demonstrated the LOF phenotype of the variants.

**Figure 3:**
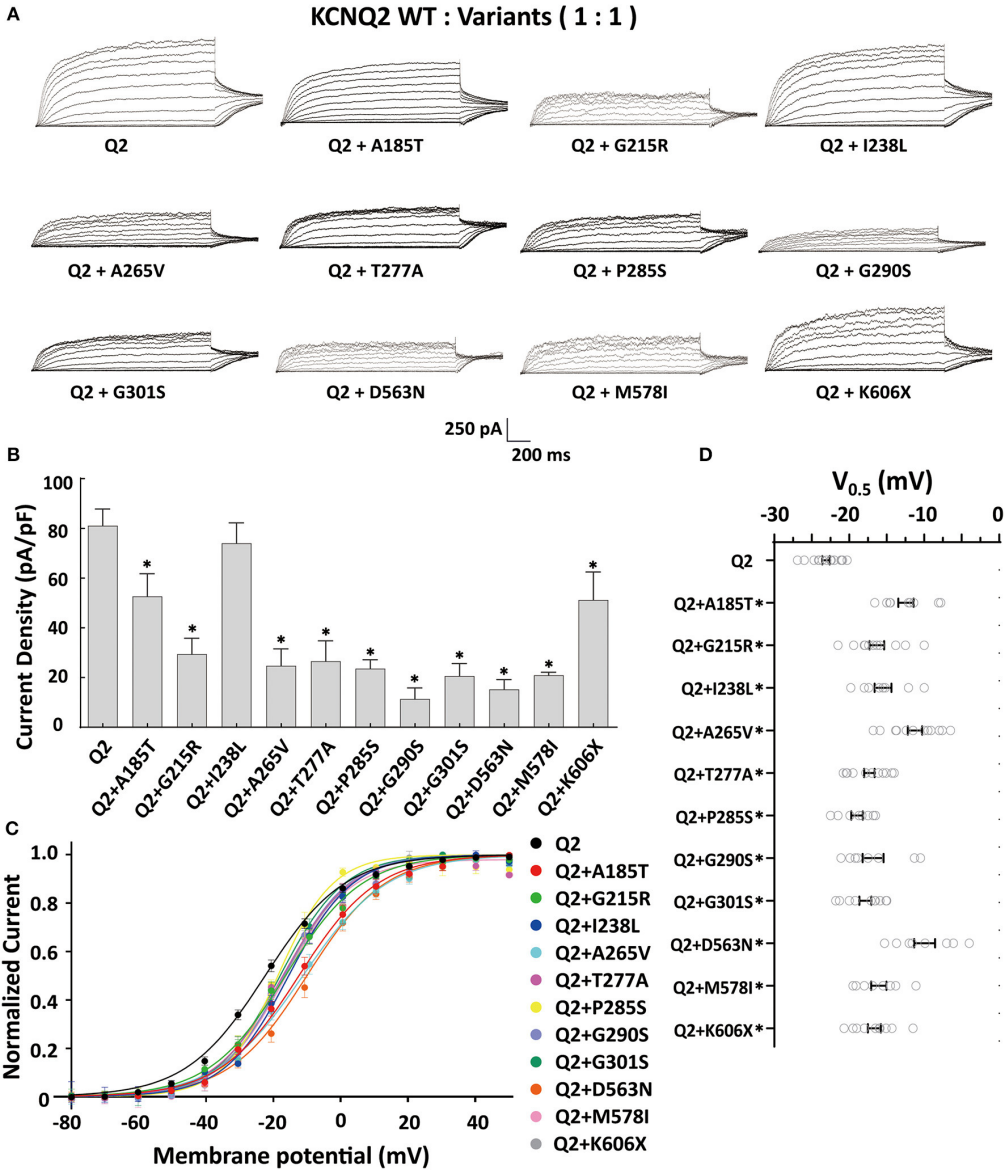
Functional properties of the KCNQ2 variants coexpressed with WTKCNQ2. **(A)** Macroscopic current traces recorded in CHO cells transfected KCNQ2 variants with WTKCNQ2in a 1:1 ratio (1.5 μg:1.5 μg). Current scale, 250 pA; time scale, 200 ms. **(B)** The mean current densities of the KCNQ2 variants coexpressed with WT in a 1:1 ratio at +40mV. **(C)** Current–voltage relationships of KCNQ2 variants coexpressed with WTKCNQ2 in a 1:1 ratio determined from tail current amplitudes. Lines represent fits of a Boltzmann function. **(D)** Differences in activation of V0.5 were determined for the KCNQ2 variants and WTKCNQ2 in a 1:1 ratio (*n* = 8–15). Statistically significant differences *p < 0.05 versus WT KCNQ2.

The tetrameric KCNQ2 channel assembled with variant and WT subunits reduced more than 50% current, not merely haploinsufficiency, namely dominate-negative effects. To evaluate these effects, we coexpressed each variant with the WT in a 1:1 ratio (1.5 ug: 1.5 ug) to simulate the heterozygous genotype (Figure 3). Compared to homomeric WT KCNQ2 (3 ug), variants p.G215R, p.A265V, p.T277A, p.P285S, p.G290S, p.G301S, p.D563N, and p.M578I were each assembled with WT in a 1:1 ratio that presented a drastically reduced current (reduced more than 50% of the relative WT channel current amplitudes), indicating a dominant-negative effect. Variants p.A185T, p.I238L, and K606X showed < 50% reduction in current when coexpressed with the WT channel, suggesting a partial current-suppressing effect without a dominant-negative effect (Figures 3A, B) (Table 2). All of the variants' heteromeric channels showed significant positive shifts in V_0.5_ compared to the WT (Figures 3C, D).

**Table 2:** Biophysical properties of mutant KCNQ2/3 channel.

	**Current density (pA/pF)**	**V_0.5_ (mV)**	**K (mV/e-Fold)**
Q2 WT	81.0 ± 6.7	−23.1 ± 0.5	11.8 ± 0.4
A185T	45.7 ± 10.2^a^	−11.8 ± 0.7^a^	11.6 ± 0.6
G215R	3.1 ± 1.2^a^	—	—
I238L	73.6 ± 8.0	−6.4 ± 1.0^a^	11.0 ± 0.8
A265V	2.0 ± 0.9^a^	—	—
T277A	5.5 ± 1.2^a^	—	—
P285S	4.0 ± 1.1^a^	—	—
G290S	3.4 ± 1.7^a^	—	—
G301S	2.3 ± 1.0^a^	—	—
D563N	6.4 ± 1.3^a^	—	—
M578I	7.2 ± 2.2^a^	—	—
K606X	2.4 ± 1.0^a^	—	—
Q2+A185T	52.0 ± 9.1^a^	−12.4 ± 1.0^a^	11.7 ± 0.6
Q2+G215R	29.4 ± 6.4^a^	−16.4 ± 1.2^a^	11.4 ± 0.5
Q2+I238L	73.9 ± 8.2	−15.5 ± 1.1^a^	10.3 ± 1.0
Q2+A265V	24.7 ± 6.9^a^	−11.2 ± 1.2^a^	12.5 ± 0.8
Q2+T277A	26.5 ± 8.2^a^	−17.3 ± 0.8^a^	10.1 ± 0.5
Q2+P285S	23.5 ± 3.6^a^	−19.0 ± 0.8^a^	8.2 ± 0.5^a^
Q2+G290S	11.3 ± 4.5^a^	−16.8 ± 1.4^a^	9.4 ± 0.6
Q2+G301S	20.6 ± 5.0^a^	−17.9 ± 0.9^a^	9.5 ± 0.6
Q2+D563N	15.1 ± 4.0^a^	−10.0 ± 1.4^a^	11.5 ± 0.6
Q2+M578I	20.8 ± 1.3^a^	−16.0 ± 1.0^a^	9.7 ± 1.0
Q2+K606X	51.1 ± 11.3^a^	−16.7 ± 0.9^a^	7.1 ± 0.6^a^
Q2 WT+Q3 WT (1:1)	281.6 ± 18.7	−24.9 ± 0.6	10.7 ± 0.4
Q2 WT+Q3 WT (0.5:1)	202.9 ± 35.3^b^	−26.3 ± 0.9	9.7 ± 0.6
Q2+A185T+Q3	200.9 ± 23.1^b^	−21.2 ± 1.0^c^	8.6 ± 0.7
Q2+G215R+Q3	139.6 ± 9.6^b^^,^^c^	−17.7 ± 0.9^b^^,^^c^	9.0 ± 1.0
Q2+I238L+Q3	270.4 ± 23.5^c^	−20.5 ± 0.8^b^^,^^c^	10.8 ± 0.6
Q2+A265V+Q3	145.2 ± 26.3^b^^,^^c^	−12.1 ± 1.2^b^^,^^c^	11.8 ± 0.9
Q2+T277A+Q3	195.3 ± 35.8^b^	−15.4 ± 0.8^b^^,^^c^	10.0 ± 1.0
Q2+P285S+Q3	205.2 ± 20.9^b^	−21.8 ± 1.0^c^	9.2 ± 0.8
Q2+G290S+Q3	71.6 ± 12.3^b^^,^^c^	−18.7 ± 0.6^b^^,^^c^	9.6 ± 0.6
Q2+G301S+Q3	191.8 ± 25.2^b^	−18.4 ± 0.7^b^^,^^c^	11.9 ± 0.5
Q2+D563N+Q3	150.5 ± 17.3^b^^,^^c^	−17.4 ± 1.0^b^^,^^c^	9.4 ± 1.0
Q2+M578I+Q3	268.2 ± 32.2^c^	−22.0 ± 0.8^c^	8.4 ± 0.8
Q2+K606X+Q3	294.3 ± 30.5^c^	−21.0 ± 0.7^b^^,^^c^	9.6 ± 0.9

Current densities were calculated as peak current at +40 mV. V_0.5_ is the voltage of half-maximal activation; k is the slope factor. Data are presented as means ± SEM, n = 8–15.

a *P* < 0.05 vs. Q2.

b *P* < 0.05 vs. Q2/Q3 (1:1).

c *P* < 0.05 versus Q2/Q3 (0.5:1).

**Figure 4:**
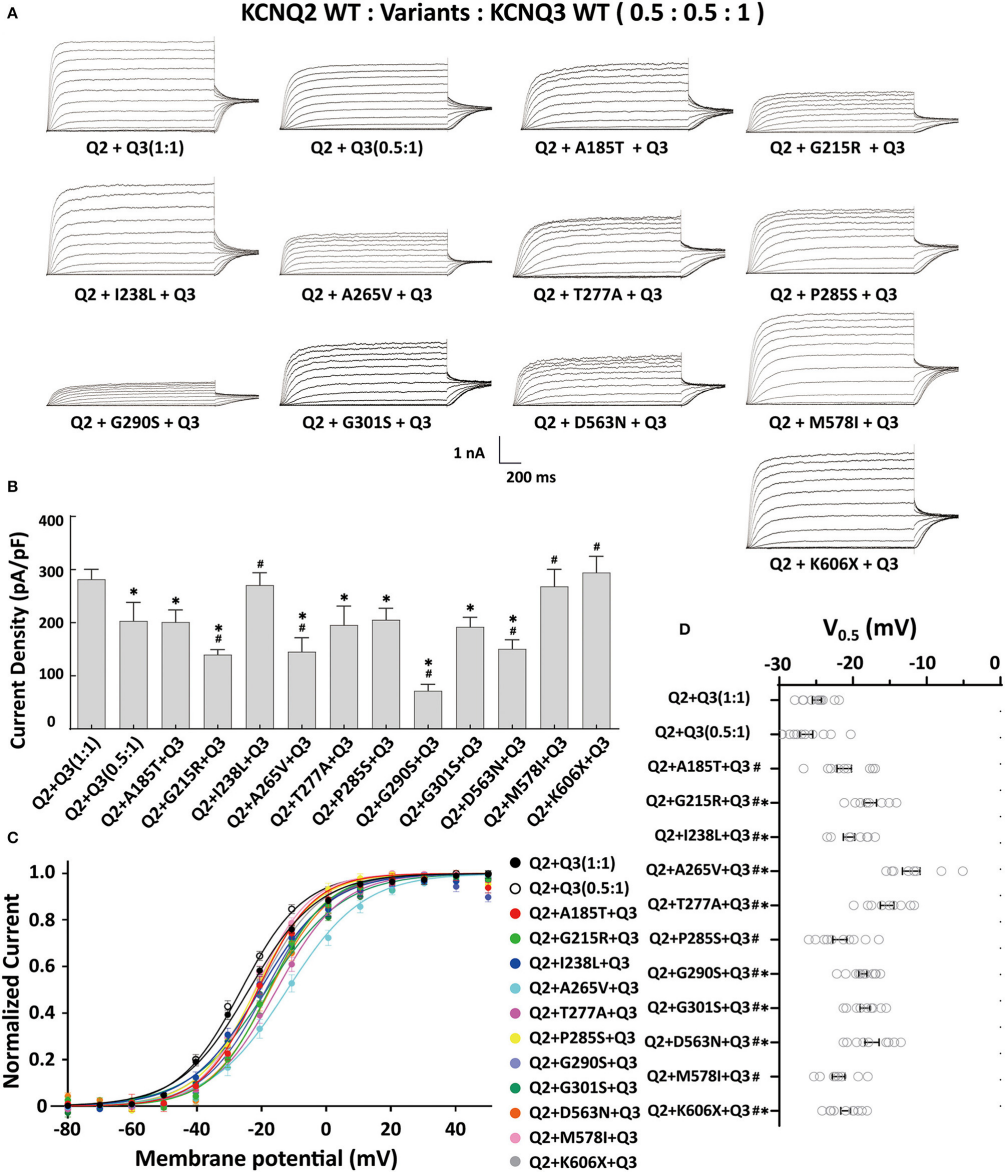
Functional properties of the KCNQ2 variants coexpressed with WTKCNQ2 and WTKCNQ3. **(A)** Macroscopic current traces recorded in CHO cells transfected KCNQ2 variants with WTKCNQ2 and WTKCNQ3 in 0.5:0.5:1 ratio (0.75 μg:0.75 μg:1.5 μg). Current scale, 1 nA; time scale, 200 ms. **(B)** The mean current densities of the KCNQ2variants coexpressed with WTKCNQ2 and WTKCNQ3 in a 0.5:0.5:1 ratio at +40 mV. **(C)** Current–voltage relationships of KCNQ2 variants coexpressed with WTKCNQ2 and WTKCNQ3 in a 0.5:0.5:1 ratio determined from tail current amplitudes. Lines represent fits of a Boltzmann function. **(D)** Differences in activation of V_0.5_ were determined for the KCNQ2 variants, WTKCNQ2 and WTKCNQ3in a 0.5:0.5:1 ratio orWTKCNQ2 and WTKCNQ3in a 0.5:1ratio (*n* = 8–11). Statistically significant differences *p < 0.05 versus WTKCNQ2/WTKCNQ3 in a 1:1 ratio. ^#^*p* < 0.05 vs. WTKCNQ2/WTKCNQ3 in a 0.5:1 ratio.

The heteromeric assembly of KCNQ2 and KCNQ3 subunits gives rise to larger currents and underlies most of the neuronal M-currents. Thus, the functional consequences of the variants were assessed in heteromeric channels formed upon coexpression of WT KCNQ2, variant KCNQ2, and WT KCNQ3 in a 0.5:0.5:1 (0.75 ug:0.75 ug:1.5 ug) ratio to simulate the heteromeric subunit composition and proportion in an affected individual (Figure 4A). Compared to their homomeric channels, most heteromeric variant channels were rescued by KCNQ3 and showed larger current sizes. Variants p.I238L, p.M578I, and p.K606X showed similar current density to the WT. The current amplitudes of p.A185T, p.T277A, p.P285S, and p.G301S were reduced by 20–30%, similar to that of cells transfected with Q2/Q3 at a 0.5:1 ratio, indicating a partial LOF. Notably, the current density in cells expressing p.G215R, p.A265V, p.G290S, and p.D563N with WT Q2/Q3 was significantly smaller than that cell expressed Q2/Q3 at a 0.5:1 ratio, therefore suggesting a strong dominant-negative effect on Q2/Q3 heteromeric channel currents exerted by the mutant subunits (Figure 3B). A significant rightward shift in the V_0.5_ was observed for p.G215R, p.I238L, p.A265V, p.T277A, p.G290S, p.G301S, and p.D563N, indicating that these variants require a more positive potential to reach their maximum currents and may not evoke enough currents to control the action potential at negative potentials (Figures 4C, D).

**Figure 5:**
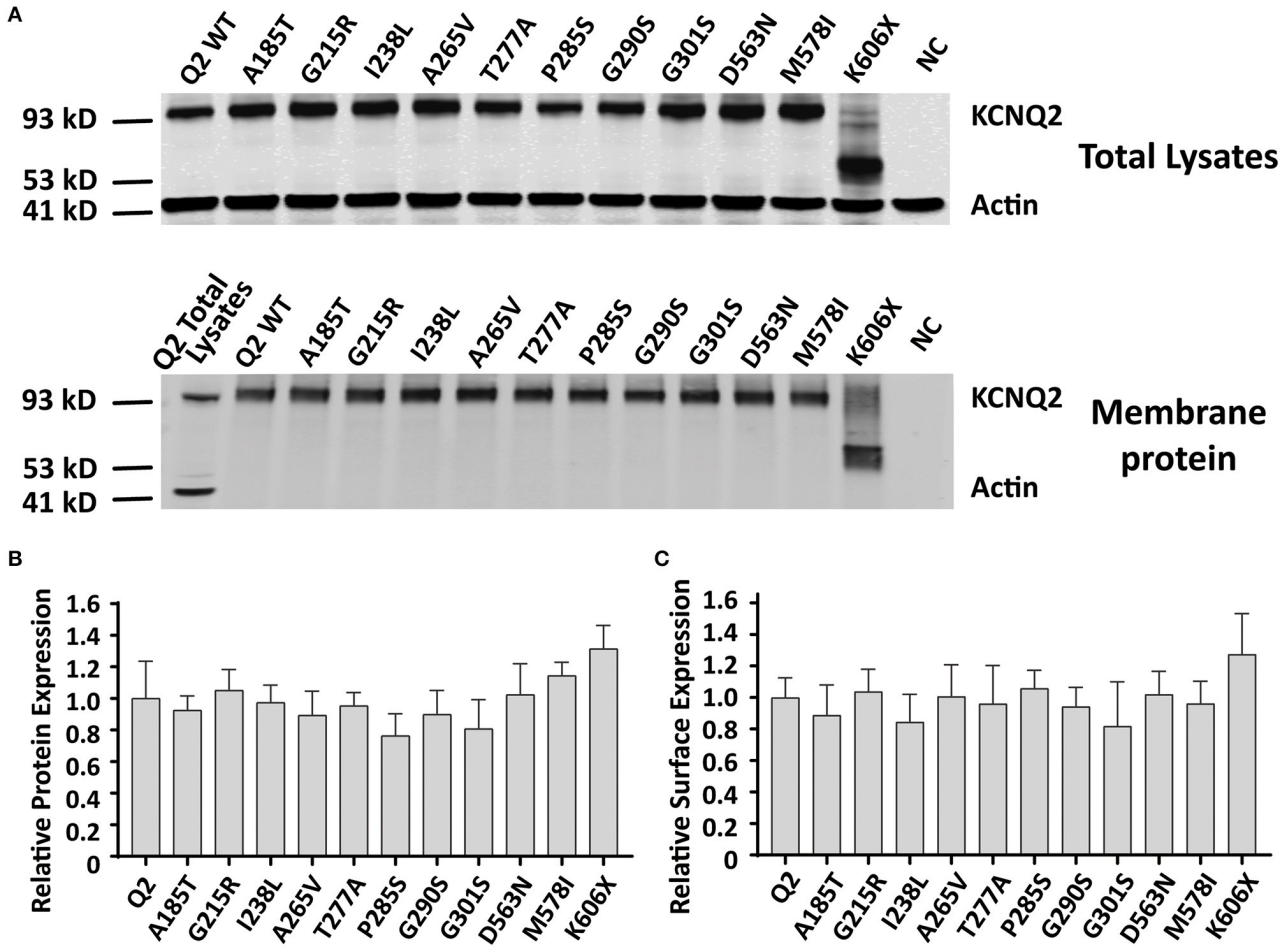
Total expression and surface expression of KCNQ2 channel. **(A)** Western blot analysis of proteins from total lysates **(top)** or plasma membrane fractions **(bottom)** from the negative control (NC, non-transfected CHO cells) or the CHO cells transfected with KCNQ2 variants. **(B)** Protein expression levels in total lysates obtained from transfected CHO cells were determined by normalizing KCNQ2 variants to the corresponding actin signals in four independent experiments. **(C)** Surface expression levels of the KCNQ2 variants normalized to WTKCNQ2 from four independent experiments. Statistically significant differences *p < 0.05 versus WT KCNQ2.

Reduced KCNQ2 variant expression or impaired trafficking to the plasma membrane may be the potential underlying mechanisms leading to the LOF effect of the KCNQ2 channel. To examine the effect of variants on the expression of KCNQ2 subunits, we used Western blots to assess the total KCNQ2 protein levels. As shown in Figure 5A, the expected 92 kDa bands were shown by using an anti-KCNQ2 antibody. The Western blot analysis of the total cell lysates from WT or variant KCNQ2 expressing CHO cells revealed that all the variants' expression levels were similar to WT (Figure 5B). Next, we investigated whether the presence of these variants was able to interfere with the trafficking of KCNQ2 subunits to the plasma membrane. The Western blot analysis of membrane protein fraction from CHO cells is presented in Figure 5C. The quantification of the bands from four different experiments showed no significant differences between WT and the variants in membrane expression levels.

## Discussion

In this study, we reported 11 *de novo* KCNQ2 variants including 10 DEE variants and 1 SeLNE variant. Among the 10 DEE variants, six of them were found to be located in the S5–S6 pore domain, which is consistent with previously identified hotspots (Goto et al., 2019). All of the DEE patients in this study we investigated showed neonatal-onset epilepsy in the first few days or weeks of birth, with tonic seizures as the initial seizure type. Despite seizure control occurring between 3 months and 9 months old, all DEE patients developed moderate-to-profound developmental delay after their first year of life.

To investigate a potential genotype–phenotype correlation, we performed functional characterization of the variants using whole-cell electrophysiological recordings from homomericKCNQ2 or heteromericKCNQ2/3 channels. Most homomeric variants showed significant LOF, with barely detectable or negligible currents. Interestingly, two novel variants, p.A185T and p.I238L, retained 56% and 91% current density relative to the WT channel, respectively. It is worth noting that both p.A185T and p.I238L significantly shifted the activation curve to more positive potentials leading to a significant current reduction in the subthreshold range of an action potential, supporting the LOF properties.

When assembled with a WT KCNQ2 subunit in a 1:1 ratio, most DEE variants showed a drastically reduced current, with over 50% reduction in relative WT channel current amplitudes, consistent with the dominant-negative effect as the primary pathogenic mechanism for the severe epileptic phenotype. Not surprisingly, the SeLNE variant K606X exhibited haploinsufficiency effects, with a 37% reduction in current amplitude. The p.A185T and p.I238L variants retained partial heteromeric channel currents when incorporated with WT KCNQ2. However, the altered gating kinetics with a significant depolarizing shift of the activation curve observed in these variants may contribute to the clinical severity of DEE. Despite not exerting dominant-negative effects, these variants might still have a detrimental impact on M-current, resulting in developmental delay and other neurologic impairments.

Most of the variants we studied were rescued by KCNQ3 and showed larger currents in the heteromeric assembly of KCNQ2 and KCNQ3 subunits. The homomeric KCNQ3 channel exhibited a more negative V0.5 (−10 to −20 mV) compared to KCNQ2 (Miceli et al., 2015; Ambrosino et al., 2018). Therefore, when combining Q2:Q3 in a 1:1 ratio, the heteromeric channel would demonstrate a negative shift in V_0.5_ relative to the homomeric KCNQ2 channel. When transfected with Q2:Q3 in a ratio of 0.5:1, where Q3 subunits constitute a larger proportion, a more pronounced negative shift in V_0.5_ would be observed compared to the heteromeric Q2/Q3 channel in a 1:1 ratio (Soldovieri et al., 2014). As a result, the V_0.5_ values of all 11 variants showed significant differences compared to Q2:Q3 in a ratio of 0.5:1. However, it is worth noting that we typically compare the differences in current density to the 0.5:1 ratio, while we rarely compare the differences in V_0.5_ to the 0.5:1 ratio. Typically, we compare the V_0.5_ values to Q2:Q3 in a ratio of 1:1. Under these conditions, variants A185T, P285S, and M578I showed no significant differences in V_0.5_ compared to Q2:Q3 in a ratio of 1:1.

Variants p.I238L, p.M578I, and p.K606X had a current density close to WT KCNQ2/3. The current amplitude of p.A185T, p.T277A, p.P285S, and p.G301S had less reduction, ~30%, similar to cells transfected with Q2/Q3 at a 0.5:1 ratio. Patients with variants p.A185T, p.P285S, and p.M578I, presenting with moderate-to-severe developmental delay, may be due to a partial loss of function (LOF) without dominant-negative effects on Q2/Q3 heteromeric channels. An mRNA molecule encoding a truncated variant may decay and fail to be translated into protein (Goto et al., 2019; Mary et al., 2021). However, our Western blot results demonstrated that the K606X variant could be translated into protein. The expression level of the K606X variant was comparable to that of the wild-type and other variants. Despite losing the intracellular helices D and the C-terminal, the K606X variant may still retain some functionality when combined with WT subunits and potentially influence the heteromeric channel, so when combining the K606X variant with Q2:Q3 in a 0.5:0.5:1 ratio, we observed a positive shift in V0.5 compared to Q2:Q3 in a ratio of 1:1 (0.01 < *p* < 0.05). However, there was no significant difference in current density compared to Q2:Q3 in a ratio of 1:1. The K606X variants did not exhibit dominant-negative effects when combined with Q2 in a ratio of 1:1 or with Q2/Q3 in a ratio of 0.5:0.5:1, which may contribute to the SeLNE phenotype. As a SeLNE variant, the patient carrying the p.K606X variant was expected to have normal development. Variants p.G215R, p.A265V, p.G290S, and p.D563N had nearly 50% reduction in current and showed dominant-negative effects in heteromeric channels when incorporated with WT Q2/Q3. Patients carrying these variants presented profound developmental impairment without even head control. p.A265 is a high-frequency mutational site located in the pore domain, and different substitutions (p.A265V, p.A265P, and p.A265T) at this site have been reported previously in patients with encephalopathy and severe or profound developmental disorders (Weckhuysen et al., 2012; Kato et al., 2013; Orhan et al., 2014). In this study, the patient with p.A265V had seizures stopped at the earliest age but had the most profound developmental delay. These results suggest that the functional consequence of the M-current of heteromericKCNQ2/3 channels caused by dominant-negative effects may be a common mechanism associated with the severity of developmental disorders in DEE. The variants with dominant-negative effects in heteromeric channels, such as p.G215R, p.A265V, and p.G290S, may be responsible for the profound developmental phenotype.

Interestingly, variants p.I238L, p.T277A, and p.G301S did not show dominant-negative effects in heteromeric channels with WTKCNQ2/Q3, while the patients carrying these variants still presented profound developmental impairment. All these three variants were located in the pore-forming domain. There were some missense mutations in this region such as KCNQ2p.Y284C, p.A294V, p.A306T, and KCNQ3 p.G310V (corresponding to the G271V mutation in KCNQ2), which showed no effects on the surface expression of the protein in the Xenopus oocyte or the CHO cell expression system (Schwake et al., 2000; Abidi et al., 2015). However, these variants were altered in total protein levels, surface expressions, or subcellular localization in hippocampal neurons (Chung et al., 2006; Abidi et al., 2015). Variants p.I238L, p.T277A, and p.G301S may also exhibit changes in the expression levels or subcellular localization in neurons and may affect motor axon excitability and transmitter release (Martire et al., 2004; Schwarz et al., 2006). The mechanisms of KCNQ2 variants associated with developmental phenotype were complex, and further studies with these variants using neuron or animal models may help us explain KCNQ2-related developmental disorders.

All variants in this study were identified as LOF, indicating a decrease in M-current amplitude from moderate to strong. This is consistent with the previous findings that most pathogenic KCNQ2 variants cause LOF and lead to severe DEE with neonatal-onset seizures (Orhan et al., 2014; Gomis-Perez et al., 2019). However, a few gain-of-function (GOF) cases have also been reported (Miceli et al., 2015; Devaux et al., 2016; Millichap et al., 2017; Xiong et al., 2022). *De novo* GOF variants in the KCNQ2 gene, such as p.R201C and p.R201H, are associated with a distinct neonatal syndrome characterized by non-epileptic myoclonus and a suppression-burst EEG pattern without seizures. On the other hand, the KCNQ2p.R198Q variant has been frequently observed in patients with West syndrome who did not have neonatal seizures. Nonetheless, all patients with GOF variants had significant developmental delays. KCNQ2/3 channels are expressed in both pyramidal neurons and interneurons (Cooper et al., 2001; Uchida et al., 2017; Springer et al., 2021). The hypothesis is that GOF variants preferentially dampen the excitability of interneurons, disrupting the balance of the excitatory network. Despite reports of homeostatic potentiation of excitatory transmission as a result of loss of KCNQ2/KCNQ3 function in interneurons, the imbalance in the excitatory network and interneurons may play a crucial role in the pathogenesis of both LOF and GOF variants in the KCNQ2 gene (Soh et al., 2018).

KCNQ3 variants affecting residues p.R230 (p.R230C, p.R230H, and p.R230S) and p.R227 (p.R227Q), which are homologous to the KCNQ2 GOF site at p.R201 and R198, respectively, are also GOF and lead to neurodevelopmental disabilities without neonatal seizures (Barro-Soria, 2019; Sands et al., 2019). Given that developmental and behavioral impairments in DEE patients exist independently of epilepsy onset, the relationship between KCNQ3 and developmental disorders is an important question to consider. While variants may alter the gating, trafficking, or subcellular localization of KCNQ2 and KCNQ3, the expression pattern of these channels in the nervous system during development may provide insight. Recent data from large transcriptomic studies and single-cell RNA sequencing indicate that the expression of both channels begins early in development and varies depending on the brain region and cell type, with KCNQ2 expression starting slightly earlier (Springer et al., 2021). Comprehensive considerations should be taken to associate the various clinical phenotypes with the ongoing exploration of mechanisms.

There are several limitations to this study. Although clinical analysis and functional characterization of 11 *de novo* KCNQ2 variants in this study suggest an association between the functional consequence and the severity of the developmental disorders, more variants need to be investigated to validate the relationships between genotypes and phenotypes in KCNQ2-related DEE. Automated patch-clamp recordings enable high-throughput evaluation of epilepsy-associated KCNQ2 variants, expanding our understanding of the molecular basis of KCNQ2-related epilepsy (Vanoye et al., 2022). Future studies using neural networks and animal models may help explain KCNQ2-related DEE from a mechanism perspective.

In summary, this study combined clinical analysis and functional characterization to investigate a group of patients carrying pathogenic KCNQ2 variants who exhibited neonatal epileptic onset and developmental delay ranging from moderate to profound. The findings support that the reduction of M-current due to dominant-negative effects underlies the mechanism of KCNQ2-related DEE. The functional impact of variants on M-current in heteromericKCNQ2/3 channels may be associated with the severity of developmental disorders in DEE, potentially serving as a predictor of neurologic prognosis.

## Data availability statement

The original contributions presented in the study are included in the article/supplementary material, further inquiries can be directed to the corresponding authors.

## Ethics statement

Ethical approval of the study was provided by the Second Affiliated Hospital of Zhejiang University (2018-080) and the study procedures have been performed in accordance with the Declaration of Helsinki and its later ethical standards. Informed consents were obtained from the parents of patients.

## Author contributions

JY, ST, and PM designed research, performed research, analyzed data, and wrote the manuscript. ZG and QS performed research and reviewed the manuscript. JF and YL drafted and critically revised the study. All authors contributed to the article and approved the submitted version.
